# ^18^FDG PET-CT imaging detects arterial inflammation and early atherosclerosis in HIV-infected adults with cardiovascular disease risk factors

**DOI:** 10.1186/1476-9255-9-26

**Published:** 2012-06-22

**Authors:** Kevin E Yarasheski, Erin Laciny, E Turner Overton, Dominic N Reeds, Michael Harrod, Steven Baldwin, Victor G Dávila-Román

**Affiliations:** 1 Department of Internal Medicine, Washington University School of Medicine, 660 South Euclid Avenue, Box 8127, St. Louis, MO, USA; 2 Department of Internal Medicine, Division of Infectious Diseases, Washington University School of Medicine, 660 South Euclid Avenue, St. Louis, MO, 63110, USA; 3 Department of Internal Medicine, Washington University School of Medicine, 660 South Euclid Avenue, Box 8031, St. Louis, MO, 63110, USA; 4 Center for Clinical Imaging Research, Mallinckrodt Institute of Radiology,, Washington University School of Medicine, 510 South Kingshighway Blvd., Box 8131, St. Louis, MO, 63110, USA; 5 Cardiovascular Imaging and Clinical Research Core Laboratory, Department of Internal Medicine, Washington University School of Medicine, 660 South Euclid Avenue, Box 8086, St. Louis, MO, 63110, USA; 6 Department of Internal Medicine, Cell Biology & Physiology, Physical Therapy, Washington University School of Medicine, Division of Metabolism, Endocrinology & Lipid Research, 660 South Euclid Avenue, Campus Box 8127, St. Louis, MO, 63110, USA

**Keywords:** Pathophysiologic molecular-level biomarker, Atherogenesis, Non-invasive imaging, Infectious disease

## Abstract

**Background:**

Persistent vascular inflammation has been implicated as an important cause for a higher prevalence of cardiovascular disease (CVD) in HIV-infected adults. In several populations at high risk for CVD, vascular ^18^Fluorodeoxyglucose (^18^FDG) uptake quantified using 3D-positron emission-computed tomography (PET-CT) has been used as a molecular level biomarker for the presence of metabolically active proinflammatory macrophages in rupture-prone early atherosclerotic plaques. We hypothesized that ^18^FDG PET-CT imaging would detect arterial inflammation and early atherosclerosis in HIV-infected adults with modest CVD risk.

**Methods:**

We studied 9 HIV-infected participants with fully suppressed HIV viremia on antiretroviral therapy (8 men, median age 52 yrs, median BMI 29 kg/m^2^, median CD4 count 655 cells/μL, 33% current smokers) and 5 HIV-negative participants (4 men, median age 44 yrs, median BMI 25 kg/m^2^, no current smokers). Mean Framingham Risk Scores were higher for HIV-infected persons (9% vs. 2%, p < 0.01). ^18^FDG (370 MBq) was administered intravenously. 3D-PET-CT images were obtained 3.5 hrs later. ^18^FDG uptake into both carotid arteries and the aorta was compared between the two groups.

**Results:**

Right and left carotid ^18^FDG uptake was greater (*P* < 0.03) in the HIV group (1.77 ±0.26, 1.33 ±0.09 target to background ratio (TBR)) than the control group (1.05 ± 0.10, 1.03 ± 0.05 TBR). ^18^FDG uptake in the aorta was greater in HIV (1.50 ±0.16 TBR) vs control group (1.24 ± 0.05 TBR), but did not reach statistical significance (*P* = 0.18).

**Conclusions:**

Carotid artery ^18^FDG PET-CT imaging detected differences in vascular inflammation and early atherosclerosis between HIV-infected adults with CVD risk factors and healthy HIV-seronegative controls. These findings confirm the utility of this molecular level imaging approach for detecting and quantifying glucose uptake into inflammatory macrophages present in metabolically active, rupture-prone atherosclerotic plaques in HIV infected adults; a population with increased CVD risk.

## Background

Atherosclerosis is initiated by a series of proinflammatory events that occur in the arterial wall causing endothelial smooth muscle disruption, macrophage activation and infiltration, oxidized lipid accumulation, and plaque formation [1-6]. Several circulating and imaging biomarkers for these proatherogenic processes have been assessed clinically [7-14]. Unfortunately, few are specific for early molecular level events involved in atherogenesis. Thus, they are not predictive biomarkers of subclinical atherosclerosis that identify people at early risk for developing vascular plaques. Moreover, none of these biomarkers provides information about the risk for plaque rupture or thrombosis that result in infarct or stroke.

People living with human immunodeficiency virus infection (HIV) have a 2-fold greater risk for experiencing a stroke or myocardial infarction than the general population [15-17]. Evidence suggests that chronic low-grade inflammation associated with the host immune response to HIV infection and ongoing viral replication contributes to greater cardiovascular disease (CVD) risk and the higher incidence of CV events in HIV infected adults [18-23]. However, the evidence is based primarily on circulating biomarkers for inflammation (hsCRP, D-dimer, cytokines) which are neither sensitive, nor specific molecular-level predictive biomarkers for early proatherogenesis or vascular plaque in/stability [21,24].

Several groups have pioneered the use of ^18^Fluoro-deoxyglucose (^18^FDG) uptake by proinflammatory macrophages present in the arterial wall as a non-invasive, sensitive, specific, and reproducible molecular level biomarker for early atheroma formation in metabolically active, rupture-prone atherosclerotic plaques [25-51]. Proinflammatory macrophages utilize glucose at a high rate [30,40,49], and 3-dimensional positron emission-computed tomography imaging (PET-CT) detects ^18^FDG uptake by macrophages in the vascular wall of animals and humans. ^18^FDG PET-CT imaging has been used to detect and quantify vascular inflammation in early atherogenesis and in vulnerable plaques in human aorta and carotid arteries, but not in people living with HIV and asymptomatic CVD.

The purpose of this proof-of-concept pilot study was to determine if ^18^FDG PET-CT imaging detects greater aortic and carotid inflammation and early atherosclerosis in HIV-infected adults with mild CVD risk and known carotid plaque than in healthy controls without significant CVD risk. If confirmed, ^18^FDG PET-CT can be used to monitor atherogenesis, determine the independent contributions of HIV infection and anti-retroviral therapy to vascular inflammation, and evaluate the anti-inflammatory, anti-atherogenic effectiveness of therapeutic interventions in people living with HIV.

### Participants

Healthy men and women were recruited from Washington University Institute of Clinical and Translational Sciences Research Participant Registry. Inclusion criteria were: 35–60 yrs old, confirmed HIV seronegative status, fasting plasma glucose <100 mg/dL and <140 mg/dL two hours after ingesting a 75-gr glucose beverage, fasting serum triglycerides <150 mg/dL, HDL-cholesterol >40 mg/dL (men), >50 mg/dL(women), resting blood pressures <130/85 mmHg), carotid intima media thickness <0.8 mm and no evidence for carotid plaque, waist circumference (at the umbilicus) <102 cm (men, <88 cm (women). Exclusion criteria were: known cardiac or cerebrovascular disease, kidney or liver disease (active hepatitis B or C), certain medications (e.g., glucose- or lipid-lowering agents, anti-hypertensives, low dose aspirin, or other anti-inflammatory agents), illegal drug use (cocaine, methamphetamines, opiates detected on urine drug screen), pregnancy, cognitive impairment that limited their ability to provide voluntary informed consent, incarcerated or otherwise unable to provide informed consent. We excluded younger adults because the prevalence of atherosclerosis is rare in people <35 yrs old. Before screening, healthy controls were informed that participation required a test for HIV-infection and the implications of a positive HIV test.

HIV infected men and women were recruited from the Washington University AIDS Clinical Trials Unit and Infectious Diseases Clinics. Inclusion criteria were: 35–60 yrs old, documented HIV seropositive status, stable antiretroviral therapy for at least the past 4 months, CD4+ T-cell count >200 cells/μL, plasma HIV RNA <50 copies/mL, fasting plasma glucose 100–126 mg/dL, or 140–200 mg/dL two hours after ingesting 75-gr glucose beverage, or fasting triglycerides >150 mg/dL, HDL-cholesterol <40 mg/dL (men), <50 mg/dL (women), or resting blood pressure ≥130/85 mmHg, carotid intima media thickness >0.8 mm or evidence of carotid plaque, or waist circumference ≥102 cm(men), ≥88 cm(women), or BMI 25–35 kg/m^2^. Exclusion criteria were identical to those used for the healthy controls. By enrolling two groups with distinctly different cardiometabolic phenotypes, we optimized our chances of detecting a difference in arterial inflammation.

We enrolled 9 HIV infected adults with cardiovascular disease (CVD) risk factors and documented (ultrasound) carotid intima media thickening or non-obstructive plaque, and 5 HIV seronegative adults with no CVD risk factors (Table 1). Right and left carotid ^18^FDG PET-CT studies were conducted on all 14 participants. Aorta ^18^FDG PET-CT studies were added after the first five participants were enrolled, so aorta ^18^FDG studies were conducted on 4 controls and 5 HIV participants. All participants provided verbal and written informed consent. The informed consent document was approved by the Human Research Protection Office and the Radioactive Drug Research Committee at Washington University School of Medicine. The study was registered with ClinicalTrials.gov (#00958815).

**Table 1:** Physical and Demographic Characteristics

**Parameter**	**Control**	**HIV+**	***p-value***
N (% female)	5 (20)	9 (11)	1.0
Ethnicity	All Caucasian	7 Cauc. 2 African American	0.51
Age (yrs)	44 ± 3 (46, 35, 51)	52 ± 3 (52, 38, 67)	0.07
# Yrs Known HIV+	0	14 ± 2 (13, 7, 20)	***<0.01***
CD4+ (cells/μL)	nd	771 ± 132 (655, 622, 1035)	
Plasma HIV RNA (copies/mL)	nd	All <50	
History Hypertension (%)	0	78	***0.02***
Family History Diabetes (%)	0	56	0.08
History Hyperlipidemia (%)	20	44	0.58
Current tobacco smoker (%)	0	33	0.26
# Yrs Smoker	0	21 ± 4 (22, 10, 30)	***<0.01***
Packs/day	0	1.4 ± 0.2 (1.3,1.0,2.0)	***<0.01***
BMI (kg/m^2^)	25 ± 1 (25, 20, 28)	29 ± 2 (26, 22, 40)	0.12
Waist circ (cm)	86 ± 3 (88, 79, 90)	101 ± 6 (95, 84, 130)	0.07
Resting Systolic BP (mmHg)	112 ± 3 (114,104,119)	129 ± 5 (128, 115, 150)	***0.01***
Resting Diastolic BP (mmHg)	71 ± 5 (72, 55, 88)	76 ± 3 (75, 66, 90)	0.48
Fasting Triglycerides (mg/dL)	63 ± 6 (60, 53, 79)	149 ± 35 (115, 43, 349)	**0.04**
HDL-cholesterol (mg/dL)	51 ± 6 (50, 37, 65)	45 ± 4 (47, 29, 68)	0.43
Total cholesterol (mg/dL)	164 ± 4 (164, 156, 172)	182 ± 9 (177, 140, 218)	0.11
Calc.LDL-cholesterol (mg/dL)	101 ± 7 (95, 91, 122)	108 ± 9 (106, 74, 156)	0.55
Framingham 10-yr Risk	2 ± 1 (1, 1, 4)	9 ± 2 (7, 1, 18)	***<0.01***
**Carotid Ultrasound**			
Mean Right & Left Posterior CIMT (mm)	0.54 ± 0.03 (0.52, 0.45, 0.63)	0.78 ± 0.02 (0.78, 0.66, 0.90)	***<0.01***
Non-obstructive Plaque	0	8 (89%)	
**Glycemic Control**			
Fasting Glucose (mg/dL)	89 ± 3 (89, 81, 96)	97 ± 4 (98, 82, 114)	0.11
Fasting Insulin (μU/mL)	2.0 ± 0.1 (2, 2, 2)	13.7 ± 4.5 (9, 6, 30)	0.06
Fasting C-peptide (ng/mL)	0.9 ± 0.1 (0.9, 0.7,1.0)	4.0 ± 0.8 (3.4, 2.3, 6.5)	***0.02***
HOMA	0.4 ± 0.0 (0.4, 0.4, 0.5)	3.2 ± 1.1 (2.6, 1.2, 7.4)	0.07
**Systemic Inflammatory Biomarkers**			
hs-CRP (mg/L)	0.8 ± 0.3 (0.8, 0.5, 1.2)	2.6 ± 2.3 (2.0, 0.6, 6.2)	0.16
D-dimer (μg FEU/mL)	0.1 ± 0.1 (0.1, 0.0, 0.2)	0.4 ± 0.5 (0.1, 0.0, 1.1)	0.27

Mean ± SE (Median, Min, Max); CIMT = carotid intima media thickness; nd = not done.

## Methods

### Carotid ultrasound imaging

Carotid artery intima-media thickness (CIMT) was measured by a single vascular sonographer using B-mode images of both carotid arteries expressed as the average thickness of the far walls of the right and left common carotid arteries; each site represents the average of 3 separate measurements [7]. The intra-class correlation coefficient for repeated measures of the CIMT is 0.91 at our laboratory [52]. The presence of carotid plaque was defined as described [7]; focal wall thickening that was ≥50% of the surrounding vessel wall or as a focal region with CIMT >1.5 mm that protruded into the lumen and was distinct from the adjacent boundary. Carotid wall thickening that did not meet this definition (<50% or <1.5 mm) was classified as a non-obstructive plaque.

### ^18^FDG PET-CT imaging

After an overnight fast, ^18^FDG (~370 MBq; 9.9 ± 0.5 mCi) was administered intravenously and 3.5 hr later, 3D-PET-CT images of the thoracic ascending, arch, and descending aorta and carotid arteries were obtained. Prior to ^18^FDG administration, fasting blood glucose concentration was measured; if >150 mg/dL the scan was rescheduled. To minimize motion artifact during image acquisition, each participant was individually fitted with a thermoplastic mask (Orfit Industries America, Jericho, NY) that was secured to the scanner table and immobilized their head, neck and shoulders.

A Biograph 40 Truepoint PET-CT scanner (Siemens, Malvern, PA) was used to acquire 3-dimensional neck and chest CT attenuation and contrast CT scans. The PET used lutetium oxyorthosilicate detectors (169 crystals/detector block, 192 detector blocks, 52 detector rings) and a 216 mm axial field of view (FOV). The CT has 40 detector rows and a 700 mm transaxial extended FOV. The CT attenuation scans used low dose CT (30 mAs (eff.), 120 kV, 0.5 sec rotation, 0.8 pitch, 28.8 mm collimation, 3 mm slice thickness, 500 mm transaxial FOV).

PET images were obtained at 2 bed positions (15 min per position) and both attenuation corrected and non-attenuation images were reconstructed in a 168 x 168 pixel matrix. Attenuation corrected PET images were reconstructed with ordered subset method of the expectation maximization (OSEM) using 4 iterations, 8 subsets and Gaussian filter 5 mm Full Width Half Maximum (FWHM). Only CT attenuated PET volumes were used for analysis. Contrast CT images were obtained immediately after the PET images were obtained. Contrast CT used 150 mAs (eff.), 120 kV, 0.5 sec rotation, 1.0 pitch, 28.8 mm collimation, 3 mm slice thickness, 500 mm transaxial FOV. Immediately prior to acquiring contrast CT images, 70 mL of Isovue-370 (Bracco Diagnostics, Princeton, NJ) were infused intravenously. The contrast agent clearly delineated the narrow carotid arteries and jugular veins, and optimized CT and PET image co-registration and analysis.

### PET-CT image analysis

Image analysis was conducted using custom extensions of MATLAB (The Mathworks Inc., Natick, MA). All images were analyzed by the same analyst (blinded to group assignment) using a standardized workflow. In general, MATLAB functions were used to co-register PET, CT attenuation and contrast CT images/datasets, quantify maximum ^18^FDG uptake (SUV) within the vessels from 1 cm above to 1 cm below the right and left carotid bifurcation, and through the ascending, arch, and descending aorta. Background ^18^FDG uptake in corresponding regions of the jugular veins and superior vena cava were used to calculate the maximum target-to-background ratio (TBR_max_) in both carotid arteries and the aorta.

Specifically, PET volumes were converted to body weight standardized uptake values (SUVbw), where, 1 mL pure water = 1gr of body weight. In both regions of interest (neck, upper chest), axial PET and CT volumes were cropped to focus and reduce the dataset sizes. Within each axial image, rigid anatomical landmarks were identified (neck = cervical vertebrae; upper chest = sternum) and used to align the contrast CT volume with corresponding PET and CT attenuation volumes. Affine volume transformation by normalized cross correlation that allowed for rotation and shift in all three planes was used to register the contrast CT scan with the CT attenuation scan using the nearest rigid (bone) landmark corresponding to the vessel of interest. The contrast CT and CT attenuation co-registration matrix obtained was then used to align the corresponding PET volume. This provided optimal PET volume co-registration with contrast CT-enhanced vascular anatomy. MATLAB functions then quantified ^18^FDG SUV metrics (mean, median, standard deviation, min, max) along the arterial and venous regions, and these were used to derive carotid and aorta TBR_max_[28].

### Serum lipids and lipoproteins

Blood was collected from an antecubital vein after a 10-12 hr overnight fast. As described [53], serum triglycerides, total- and HDL-cholesterol were measured and LDL-cholesterol estimated in the Core Laboratory for Clinical Studies at Washington University Medical Center. The accuracy of these methods is verified and standardized by participation in the Centers for Disease Control (CDC) Lipid Standardization Program, the CDC Cholesterol Reference Method Laboratory Network, and the College of American Pathologists external proficiency program [54]. Framingham 10-yr coronary heart disease (CHD) risk was determined using an American Heart Association calculator (http://hp2010.nhlbihin.net/atpiii/calculator.asp?usertype=prof).

### Blood hormones and metabolites

As described [55], plasma glucose concentration was quantified using an automated YSI glucose analyzer (Yellow Springs Instruments, Yellow Springs, OH), and plasma insulin and C-peptide concentrations were determined using chemiluminescent immunometric methods (Immulite; Siemens, Los Angeles, CA). The homeostasis model assessment of insulin resistance (HOMA) was calculated as described [56].

### Systemic inflammatory biomarkers

D-dimer and highly sensitive C-Reactive Protein (hsCRP) concentrations were quantified using particle enhanced immuno-turbidimetric assay kits according to manufacturer’s instructions (Roche Diagnostics, Indianapolis, IN). Human plasma CRP and D-dimer complex with latex particles coated with a monoclonal antibody directed against CRP or D-dimer epitopes, and the precipitate was assayed for turbidity on a Roche/Hitachi Cobas c system. Adequate amounts of archived plasma from 4 healthy controls and 5 HIV participants were available for these assays.

### Data analysis

Mean ± standard error (SE), median, minimum and maximum values are reported for the participant characteristics. Physical and demographic characteristics were compared using a non-parametric Fishers’ exact test or Kruskal–Wallis one-way analysis of variance by ranks test. Carotid and aorta maximum TBR_max_ were compared between groups using an unpaired *t*-test. P < 0.05 was accepted as significant.

## Results

The two groups had similar demographic characteristics (age, sex, ethnicity; Table 1). Mean and median age for HIV + participants (Mean ± SE; 52 ± 3 yrs, median = 52 yrs) were numerically, but not statistically higher (p = 0.07) than controls (44 ± 3 yrs, median =46 yrs). Immune (CD4+ T-cell count = 771 ± 132 cells/μL) and virologic (all <50 copies HIV RNA/mL) status were stable and controlled in the HIV + participants. By design, cardiovascular disease risk profiles were worse among HIV + participants than controls (Table 1). The Framingham 10-yr coronary heart disease (CHD) risk score was greater (9 ± 2%) in HIV + participants than in controls (2 ± 1%; p < 0.01), but by definition these represent low (<10%) risk for CHD events (MI, stroke). Four of the 9 HIV participants had Framingham 10-yr CHD risk scores between 10-20%. The higher CHD risk score among HIV + participants was attributed to current tobacco use and higher systolic blood pressure (history of hypertension).

The mean intima media thickness of the common carotid arteries was greater (p < 0.01) in HIV + participants (0.78 ± 0.02 mm) than controls (0.54 ± 0.03 mm), and eight of nine HIV + participants had ultrasound detectable non-obstructive plaques in at least one carotid artery, while no plaques were detected in controls. Glycemic control parameters (fasting glucose, insulin, C-peptide, HOMA) were not different between control and HIV + participants. Fasting triglycerides were greater (p = 0.04) in HIV + participants (149 ±35 mg/dL) than controls (63 ± 6 mg/dL), but total-, HDL-, and calculated LDL-cholesterol levels were not different between groups. D-dimer and hsCRP levels were numerically, but not statistically (P > 0.16), higher in HIV + participants than controls.

**Figure 1:**
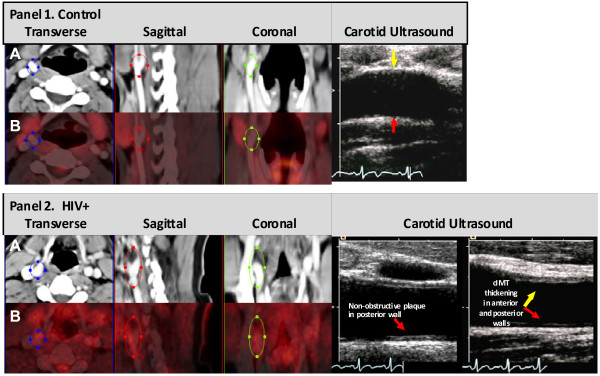
**Representative co-registered 3-dimensional positron emission (PET) and contrast-enhanced computed tomography (CT) images of the right carotid artery of a healthy HIV-seronegative control male (Panel 1), and an HIV infected man with CVD risk factors (Panel 2).** Transverse, sagittal, and coronal contrast CT images (A) and PET ^18^FDG uptake images (B) along with the corresponding carotid ultrasound images for these two men are shown. The anterior wall of the right carotid artery (upper portion of the carotid ultrasound image) is indicated with a yellow arrow and the posterior wall with a red arrow (lower portion of the image). In the HIV infected man (Panel 2), ultrasound imaging detected increased carotid artery intima media thickness in both the anterior and posterior walls and a non-obstructive plaque in the posterior wall of the right carotid artery. In the healthy control male (Panel 1), the intima media thickness was normal and no plaques were present in the anterior or posterior walls of the right carotid artery. Carotid PET imaging detected regions of higher ^18^FDG uptake (red nodules in blue, red and green ovals) in the HIV infected man (Panel 2B), while less ^18^FDG uptake was detected in the carotid artery of the healthy control male (Panel 1B).

**Figure 2:**
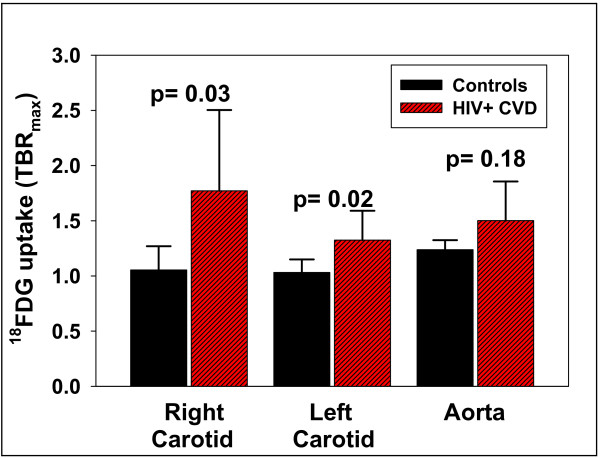
**Right and left carotid**^**18**^**FDG uptake (Mean ± SE) was greater (*P*** **< 0.03) in the HIV group (n = 9; 1.77** ± **0.26, 1.33** ± **0.09 target to background ratio-max (TBR**_**max**_**)) than in the control group (n = 5; 1.05** ± **0.10, 1.03** ±**0.05 TBR**_**max**_**).** Aorta ^18^FDG uptake tended (*P* = 0.18) to be greater in HIV (n = 5; 1.50 ±0.16 TBR_max_) vs control group (n = 4; 1.24 ±0.05 TBR_max_).

Representative images of carotid ^18^FDG PET uptake superimposed on the contrast CT images, and the corresponding carotid ultrasound images are provided for an HIV + and a control participant (Figure 1). Right and left carotid ^18^FDG uptake (TBR_max_) was greater (*P* < 0.03) in HIV + participants (1.77 ±0.26, 1.33 ±0.09) than controls (1.05 ± 0.10, 1.03 ± 0.05; Figure 2). Aorta ^18^FDG TBR_max_ tended (*P* = 0.18) to be greater in HIV + participants (1.50 ± 0.16) than controls (1.24 ±0.05; Figure 2).

## Discussion

Carotid artery ^18^FDG PET-CT imaging detected differences in vascular inflammation and early atherosclerosis between HIV-infected adults with CVD risk factors and healthy HIV-seronegative controls. These findings confirm the utility of this molecular level imaging approach for detecting and quantifying glucose uptake into inflammatory macrophages present in metabolically active, rupture-prone atherosclerotic lesions or early non-obstructive plaques in HIV infected adults; a population with increased CVD risk.

Vascular inflammation has been implicated in the underlying pathophysiology for the higher incidence of myocardial infarction and stroke in HIV infected adults taking anti-retroviral medications. However, this suggestion is based on indirect, non-specific measures of circulating pro-inflammatory or pro-oxidant stress biomarkers, or static vascular imaging methods (carotid intima media thickness, coronary calcium deposition). We provide the first direct, molecular-level evidence for the presence of metabolically active, inflammatory vascular lesions/plaques in people living with HIV infection. It is important to note that the HIV participants studied had modest clinical evidence of CVD risk; characterized by carotid intima media thickening or small non-obstructive plaques and low Framingham 10-yr CHD risk profiles (9 ± 2% risk). However, carotid ^18^FDG PET-CT imaging and greater TBR_max_ provided clearer evidence of atherosclerotic vascular disease in HIV + participants.

We specifically selected participants with ultrasound-detected carotid intima media thickening and non-obstructive plaques in order to assess the ability of vascular ^18^FDG PET-CT imaging to detect an expected difference in vascular inflammation between healthy controls and HIV-infected men and women with CHD risk. In agreement with others, we found that not all calcified lesions are metabolically active, inflammatory, rupture-prone plaques [25,26]. The current study was underpowered to definitively assess the relationship between ^18^FDG uptake and carotid thickness. The relationship between plaque morphology and ^18^FDG uptake should be further investigated in future studies.

This imaging method may be useful for addressing several critically important clinical questions in HIV infected people, e.g., Is there vascular inflammation in untreated HIV-infection? Does highly active anti-retroviral therapy reduce or worsen vascular inflammation? Is vascular inflammation worse in older HIV-infected adults than in age- and CVD risk factor-matched HIV-seronegative adults? Do effective treatments for insulin resistance, dyslipidemia, or anti-inflammatory agents reduce vascular inflammation in HIV? In the long-term, does a reduction in vascular inflammation translate to fewer clinical events (stroke, MI) in HIV? This non-invasive method is ideal for examining interactions among vascular inflammation and CVD risk factors (insulin resistance, central obesity, dyslipidemia) on early atherosclerotic progression in HIV and other autoimmune disorders where inflammatory stimuli are implicated (e.g., systemic lupus erythematosus, rheumatoid arthritis, Crohn’s disease).

Circulating inflammatory biomarker levels (hsCRP, D-dimer) were variable, but on average, were 3–4 times higher in HIV than healthy controls. This supports the generalization that even well-controlled HIV (using contemporary anti-viral agents) is associated with a chronic, low-grade, pro-inflammatory state, but the stimulus, source, location and severity of the inflammation cannot be discerned from these plasma biomarkers. HIV related inflammation can be caused by multiple factors, including chronic replicating virus, anti-HIV medications, gut microbial translocation, obesity, diabetes, tobacco/alcohol/illegal drug abuse, hepatitis co-infection, or other co-morbidities. ^18^FDG PET-CT specifically revealed vascular inflammation in the carotid arteries as a quantifiable source for molecular-level, pro-inflammatory events that are biochemically related to early atherogenesis, and if left unrestrained, can precipitate a CV event.

This study has limitations. We had a small sample size and we may have been underpowered to detect certain between group differences. But, these are expensive imaging studies, and therefore we intended these data to show proof-of-principle for larger clinical studies. On average, the HIV infected adults tended to be older than the healthy controls. Advanced age is associated with more vascular inflammation and ^18^FDG uptake. But, the focus of this study was not on “what causes vascular inflammation in HIV?” Instead, the focus was on a dichotomous outcome; i.e., can we detect vascular inflammation using ^18^FDG uptake in HIV with mild CVD risk? The intent was not to address the question “is vascular inflammation worse in age-matched HIV vs healthy controls?” This is an excellent follow-up study, now that we have developed the technique and we understand the usefulness of ^18^FDG PET-CT imaging for detecting early, low-level vascular inflammation in people living with HIV. We did not attempt to quantify ^18^FDG uptake in the coronary vessels; these are very narrow, in motion, and surrounded by the glucose-consuming heart muscle. Attempts to image inflammation in the coronary vessels using ^18^FDG have been made [57]. We cannot determine the specific risk factor that caused greater ^18^FDG uptake in HIV participants (e.g., tobacco use, hypertension, glycemic control, higher triglycerides) because the two groups were selected based on their distinctly different cardiometabolic phenotypes. Likewise, the study was not designed to determine whether HIV-infection *per se,* or which specific anti-HIV medication caused greater ^18^FDG uptake in the HIV + participants. However, these pathogenesis questions can be addressed given the proof-of-principle findings reported here. Indeed, recent studies have begun to investigate these questions using ^18^FDG PET-CT [58].

## Conclusion

Carotid ^18^FDG PET-CT imaging detected significant vascular inflammation in HIV- infected men and women with low Framingham CHD risk scores, suggesting that this molecular imaging method is sensitive to early pro-atherosclerotic processes in a clinical population suspected of having chronic, low-grade inflammation-induced cardiovascular disease.

## Abbreviations

3D, Three dimensional; AIDS, Acquired immunodeficiency syndrome; BMI, Body mass index; CDC, Centers for disease control; CHD, Coronary heart disease; CIMT, Carotid intima media thickness; cm, centimeters; CT, Computed tomography; CV, Cardiovascular; CVD, Cardiovascular disease; eff, Effective; 18FDG, 18Fluoro deoxy-glucose; FOV, Field of view; FWHM, Full width half maximum; HDL, High density lipoprotein; HIV, Human immunodeficiency virus; HOMA, Homeostasis model assessment of insulin resistance; hr, hours; hsCRP, Highly sensitive c-reactive protein; kg, kilograms; kV, kilovolts; m, meters; mAs, milliamperes; MBq, megabecquerel; mCi, milliCuries; mg/dL, milligrams per deciliter; μg FEU/mL, Micrograms of human fibrinogen equivalent units per milliliter; MI, Myocardial infarction; min, minutes; mL, milliliters; μL, microliters; mm, millimeters; mmHg, millimeters mercury; μU/mL, microUnits per milliliter; ng/mL, nanograms per milliliter; OSEM, Ordered subset method of the expectation maximization; PET, Positron emission tomography; RNA, Ribonucleic acid; SE, Standard error; sec, seconds; SUV, Standard uptake value; TBR, Target to background ratio; yrs, years.

## Competing interests

The authors declare that they have no competing interests.

## Authors’ contributions

KEY conceived, designed, and coordinated the study, acquired and interpreted data, performed the statistical analyses, and drafted the manuscript. EL participated in the design and coordination of the study, and acquired data. ETO and DNR participated in the design and coordination of the study, acquired and interpreted data, monitored participants, and helped draft the manuscript. MH and SB participated in the design and coordination of the study, acquired PET-CT images, assisted with PET-CT image analysis, and helped draft the manuscript. VGD-R participated in the design and conduct of the study, acquired, analyzed, interpreted carotid ultrasound images, and helped draft the manuscript. All authors read and approved the final manuscript.
